# Alterations in the vitamin D endocrine system during pregnancy: A longitudinal study of 855 healthy Norwegian women

**DOI:** 10.1371/journal.pone.0195041

**Published:** 2018-04-11

**Authors:** Miriam K. Gustafsson, Pål R. Romundstad, Signe Nilssen Stafne, Anne-Sofie Helvik, Astrid Kamilla Stunes, Siv Mørkved, Kjell Åsmund Salvesen, Per Medbøe Thorsby, Unni Syversen

**Affiliations:** 1 Department of Public Health and Nursing, Faculty of Medicine and Health Sciences, Norwegian University of Science and Technology (NTNU), Trondheim, Norway; 2 Division of Mental Health Care, Trondheim University Hospital (St Olavs hospital), Trondheim, Norway; 3 Clinic of Clinical Services, Trondheim University Hospital (St Olavs hospital), Trondheim, Norway; 4 Department of Clinical and Molecular Medicine, Faculty of Medicine and Health Sciences, Norwegian University of Science and Technology (NTNU), Trondheim, Norway; 5 Trondheim University Hospital (St Olavs hospital), Trondheim, Norway; 6 Department of Obstetrics and Gynaecology, Trondheim University Hospital (St Olavs hospital), Trondheim, Norway; 7 Hormone Laboratory, Department of Medical Biochemistry, Oslo University Hospital, Aker sykehus, Oslo, Norway; 8 Department of Endocrinology, Trondheim University Hospital (St Olavs hospital), Trondheim, Norway; Garvan Institute of Medical Research, AUSTRALIA

## Abstract

To ensure optimal calcium accrual in the fetal skeleton, a substantial rise occurs in 1,25-dihydroxyvitamin D (1,25(OH)_2_D), but is dependent on sufficient 25-hydroxyvitamin (25(OH)D). Large longitudinal studies addressing free 25(OH)D and 1,25(OH)_2_D during pregnancy are scarce. We aimed to assess levels of and relationship between 25(OH)D, 1,25(OH)_2_D, vitamin D-binding protein (DBP), parathyroid hormone (PTH), and free 25(OH)D during pregnancy; determinants of vitamin D status; and association between vitamin D indices or PTH and pregnancy outcomes (gestational diabetes mellitus and birthweight). Altogether 855 pregnant Norwegian Caucasian women from Trondheim and Stavanger (latitude 63°N and 58°N) were recruited; 94 were lost to follow-up. The study was originally a randomized controlled trial (2007–2009) with gestational diabetes as primary outcome. Data were collected in second and third trimester. In third trimester, 246 (34%) had vitamin D insufficiency and 52 (7%) deficiency (25(OH)D <50 and <30nmol/L, respectively). During wintertime in third trimester, 61 (47%) from Trondheim and 23 (51%) from Stavanger exhibited vitamin D insufficiency. PTH was elevated in 27 (3.7%). Estimate of change between trimesters was (95% CI): 25(OH)D -1.8 (-2.8 to -0.7) nmol/L, DBP 0.62 (0.57 to 0.66) μmol/L, calculated free 25(OH)D -1.7 (-2.0 to -1.4) pmol/L, PTH 0.81 (0.72 to 0.90) pmol/L, 1,25(OH)_2_D (sub-analysis) 31.4 (CI 24.7 to 38.2) pmol/L. A decrease in 1,25(OH)_2_D occurred in 45% of those with vitamin D deficiency, and they also exhibited lower levels than women with adequate vitamin D status. No association of vitamin D indices and PTH with pregnancy outcomes was observed. Women in Trondheim displayed lower 25(OH)D levels, despite minor latitudinal differences. Less than one-fifth adhered to the authorities’ vitamin D recommendations. These findings demonstrate that hypovitaminosis D is prevalent among pregnant women living in northern latitudes, especially during the dark season, and there is an unmet need to ensure adequate vitamin D intake.

## Introduction

Vitamin D (vitD) inadequacy among pregnant women is prevalent worldwide, and has been associated with adverse pregnancy outcomes [1–10]. Developmental origins of disease have gained increasing attention, and maternal hypovitaminosis D during fetal life is one of the factors suggested to be of significance for future disease, including osteoporosis and cardiovascular disease [11–13].

Ultraviolet-B (UVB) radiation of the skin is considered to be the major determinant of vitD levels [3, 8, 14]. However, due to latitude, cutaneous synthesis of vitD occurs less than six months of the year in the Nordic countries, and dietary content is also limited [9, 14–17]. 25-hydroxyvitamin D (25(OH)D) is metabolized in the kidneys by 1-alpha-hydroxylase to the active form, 1,25-dihydroxyvitamin D (1,25(OH)_2_D) [3, 4, 18]. Through the vitamin D receptor, regulates 1,25(OH)_2_D hundreds of genes in a variety of body tissues [6, 14, 19, 20]. A major proportion of 25(OH)D and 1,25(OH)_2_D binds to vitamin D-binding protein (DBP) or albumin (>99%) [21, 22]. Free and bioavailable fractions seem to be more strongly correlated to the biological activity [14, 22–24]. Serum levels of 25(OH)D are used for evaluation of vitD status [3, 14, 15, 21]. The optimal levels of 25(OH)D and 1,25(OH)_2_D during pregnancy are, however, not settled, and recommendations concerning vitD intake are diverging [4–6, 14, 17, 25].

In pregnancy, the calcium requirement of the fetus results in profound changes in maternal calcium homeostasis [4, 26]. Whereas parathyroid hormone (PTH) plays a major role in calcium and bone metabolism in the non-pregnant state, vitD appears to be a prominent regulator during pregnancy [8, 27]. This is reflected in a two- to threefold rise in 1,25(OH)_2_D levels to increase intestinal calcium absorption, and ensure mineralization of the fetal skeleton [4, 26, 27]. This rise is dependent on sufficient 25(OH)D [28]. The relationship between 25(OH)D and 1,25(OH)_2_D during pregnancy remains, however, unclear [18, 23, 26]. There are many determinants of 25(OH)D contributing to the diverse prevalence rates reported during pregnancy [3, 5, 9, 16, 29, 30]. A large variation in 1,25(OH)_2_D levels in third trimester has also been observed [18]. Larger longitudinal studies concerning 1,25(OH)_2_D, free and bio-available vitamin D in pregnancy are scarce [18, 21, 23].

We aimed to assess 25(OH)D, 1,25(OH)_2_D and PTH levels and the relationship between these parameters among Caucasian women in the two final trimesters. DBP was analyzed for calculation of free and bioavailable 25(OH)D and free 1,25(OH)_2_D. Furthermore, we aimed to investigate determinants of vitD, including seasonal and latitudinal variation, and adherence to the recommendations on vitD and calcium intake. Finally, we wanted to explore the association of total and free 25(OH)D, 1,25(OH)_2_D and PTH levels with pregnancy outcomes (gestational diabetes mellitus (GDM) and birthweight).

## Materials and methods

### Study design and population

**Fig 1:**
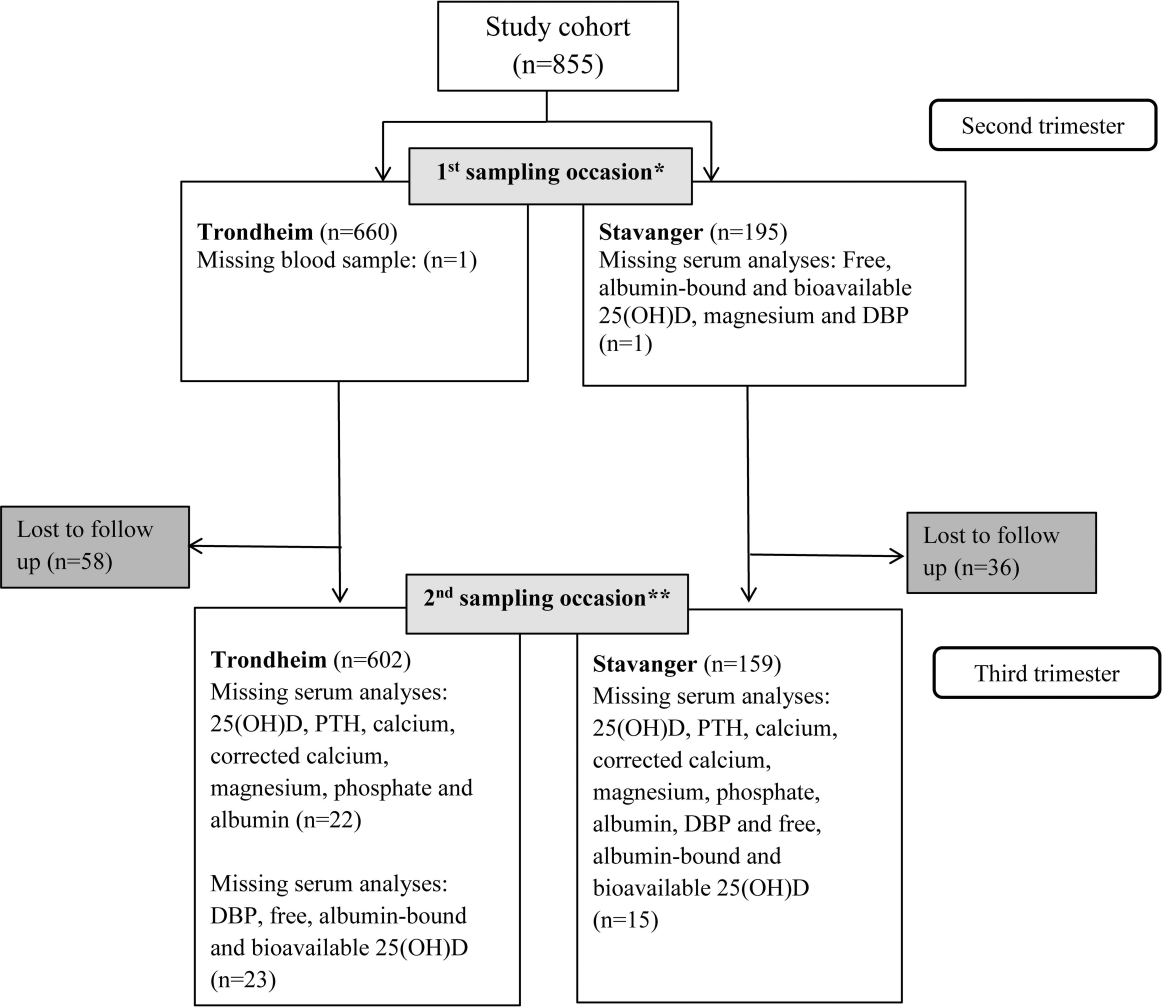
Flow diagram of the study population. *Between 18 to 22 weeks of pregnancy. **Between 32 to 36 weeks of pregnancy. Abbreviations: DBP, Vitamin D-binding protein; PTH, parathyroid hormone.

The current study included 855 pregnant Norwegian women from the cities of Trondheim (n = 660), latitude 63°N and Stavanger (n = 195), latitude 58°N (Fig 1). They participated originally in a randomized controlled trial (RCT), conducted between 2007 and 2009, aiming to investigate the antenatal health effects of an exercise program, and the primary outcome was gestational diabetes mellitus [31]. The present study was a secondary analysis of the RCT. Healthy Caucasian women, 18 years and older, with a singleton live fetus were included. Exclusion criteria were high-risk pregnancies and diseases that could hinder participation [31]. The two groups were homogenous at inclusion and after the intervention, and were merged in the current study.

Serum 25(OH)D levels <50 nmol/L were classified as insufficiency and 25(OH)D levels <30 nmol/L as deficiency (VDD) according to the US Institute of Medicine (IOM) and Nordic Nutrition recommendations [17, 25, 32]. The seasons were divided as follows: winter (December-February), spring (March-May), summer (June-August) and autumn (September-November). Gestational hypertension was defined as systolic blood pressure (BP) >140 mm Hg, diastolic BP >90 mm Hg, or both in women with no pregestational hypertension. The criteria for GDM were fasting glucose level in whole blood ≥6.1 mmol/L, or plasma glucose ≥7.0 mmol/L, or 2-hour glucose level ≥7.8 mmol/L after oral glucose tolerance test in women with no pregestational diabetes mellitus [31].

The study was conducted in accordance with the ethical principles in the declaration of Helsinki, approved by the Regional Committee for Medical and Health Research Ethics (REK 4.2007.81) and registered in the ClinicalTrials.gov (NCT 00476567). Written informed consent was obtained from all participants.

### Data collection

The participants were recruited consecutively, and clinical data and blood samples were collected in second and third trimester (pregnancy week 18–22 and 32–36) [31]. Questionnaires regarding sociodemographic variables, diet and supplements, childbirths, medical history, smoking behavior and physical activity were completed. A sub-analysis of circulating 1,25(OH)_2_D was performed in 250 women from Trondheim. To ensure that participants with both low and high levels of 25(OH)D were included in the subgroup analysis of 1,25(OH)_2_D, the serum levels of 25(OH)D in third trimester were divided into five categories: ≤30 nmol/L, 25(OH)D >30 to ≤50 nmol/L, 25(OH)D >50 to ≤75 nmol/L, 25(OH)D >75 to ≤100 nmol/L and 25(OH)D >100 nmol/L. From each category of 25(OH)D, 50 women were sampled for analysis of 1,25(OH)_2_D in second and third trimester. We have applied probability weights (the inverse of the probability of an observation being selected into the sample) in the statistical analyses of 1,25(OH)_2_D to produce estimates representative of the total Trondheim population [33, 34]

### The Food Frequency Questionnaire

A self-administered optical mark readable Food Frequency Questionnaire (FFQ) containing around 180 food items was used to collect information about vitD and calcium intake [35, 36]. The serving size alternatives were specified in household units, and calculated in grams using a software developed at the Institute for Nutrition Research, University of Oslo [35]. The FFQ was established for dietary surveys in the general Norwegian population age 16 to 67 years, and has been validated [35, 37].

### Serum analyses

Blood samples were collected after fasting, and the sera were stored at -80°C. The following analyses were conducted at Trondheim University Hospital: 25(OH)D and PTH by electrochemiluminescence immunoassay (ECLIA), calcium by a colorimetric method, and magnesium, phosphate, albumin and creatinine by photometric methods. All assays were delivered by Roche Diagnostics Ltd., Switzerland. Total calcium was corrected for the albumin concentration [38]. DBP and 1,25(OH)_2_D were analyzed at the Hormone Laboratory, Oslo University Hospital; DBP by an in-house competitive radioimmunoassay with GC-globulin (Sigma-Aldrich Corp, St. Louis, MO, USA) and polyclonal anti-GC-globulin antibodies (DakoCytomation, Glostrup, Denmark), and 1,25(OH)_2_D by an enzyme immunoassay (IDS Nordic A/S immunodiagnosticsystems) [39, 40]. Reference range, limit of detection and coefficient of analytical variation (CV) for the different analyses are presented in S1 Table.

### Calculation of free and bioavailable 25(OH)D and free 1,25(OH)_2_D

We compared two methods reported by Vermeulen et al. and Bikle et al. for calculation of free 25(OH)D, and the estimates were similar (difference of ~1%) [41–43]. Free 25(OH)D and 1,25(OH)_2_D are presented according to Bikle et al. [41, 42, 44]:

$$D_{free}=\frac{D_{total}}{\left(1+([binding\;constant\;albumin]\times albumin)+([binding\;constant\;DBP]\times DBP)\right)}$$

D_free_ is the calculated free levels of 25(OH)D or 1,25(OH)_2_D. D_total_ is the total serum levels of 25(OH)D or 1,25(OH)_2_D. Albumin-bound 25(OH)D (D_alb_) was calculated as follows [39, 42]:

$$D_{alb}=(free\;25(OH)D\times [binding\;constant\;albumin]\times albumin)$$

The binding constant was 6 x10^5^ M^-1^ between 25(OH)D and albumin, and 5.4 x 10^4^ M^-1^ between 1,25(OH)_2_D and albumin. The binding constant was 7 x 10^8^ M^-1^ between 25(OH)D and DBP, and 3.7 x 10^7^ M^-1^ between 1,25(OH)_2_D and DBP [41, 42, 44]. Bioavailable 25(OH)D was calculated as the sum of albumin-bound and free 25(OH)D [42]. The percentage of free 25(OH)D was estimated as [23]:

$$\left(\frac{free\;25(OH)D}{total\;25(OH)D}\right)\;x\;100$$

### Statistical analyses

SPSS statistics Version 22.0 (Armonk, NY: IBM Corp) and Stata version 13 (StataCorp LP, College Station, TX, USA) were used for the statistical analyses. In general data are presented as the arithmetic mean with standard deviation (SD) or 95% confidence intervals (CI).

A mixed model with fixed effects (streg with fe option) was used in Stata to study within-subject variations of vitamin D and related measures from second to third trimester [45]. The model included a random intercept. To account for the considerable variation in exposure to sunlight over the year and increase the precision, we adjusted for season. Model-based serum levels in second and third trimester were estimated by using the postestimation command lincom (linear combinations of estimators). Each season was given a weight of 0.25 and this approach gave an estimate covering each season with similar weights.

To evaluate differences between study sites by season, we used multivariable linear regression and did separate analyses for second and third trimester. In these analyses, we adjusted for season, age, pre-pregnancy BMI, parity and pre-pregnancy physical activity. When we performed analyses for the third trimester, we also adjusted for group randomization (from the in the original RCT) to take potential treatments effects into account. In the model, we allowed for interaction between study site and season, and used likelihood ratio test to assess possible interactions. In additional analyses we adjusted for vitamin D supplementation and education.

The model-based serum levels in Trondheim and Stavanger and the seasonal serum levels at both study sites were estimated using the postestimation command lincom. In the model, each season was given a weight of 0.25. Furthermore, pre-pregnancy physical activity was given a weight of 0.71 (based on the proportion of women that were exercising regularly before pregnancy), and parity 0.43 (based on the proportion of women with one or more children). Continuous variables (pre-pregnancy BMI and age) were mean-centered.

Linear and logistic regression analyses were used to estimate the potential association between serum levels of total 25(OH)D, calculated free 25(OH)D, 1,25(OH)_2_D and PTH levels in second trimester and pregnancy outcomes (GDM and birthweight (BW)). The same analyses were also performed in third trimester. In the multivariable regression models, we adjusted for study site, season, age, pre-pregnancy BMI, parity and pre-pregnancy physical activity. In additional analyses, we also adjusted for education, and intake of vitamin D, calcium and fish.

To assess the association between total 25(OH)D and 1,25(OH)_2_D we used simple linear regression. In all analyses involving 1,25(OH)_2_D, we used the pweight function in Stata to account for the sampling scheme (the inverse of the probability of an observation being selected into the sample). In this study we did not make any adjustment for multiple testing.

## Results

### Population

The participants from Trondheim and Stavanger were homogeneous in terms of baseline characteristics (Table 1). Prenatally, 17 (2.0%) were underweight (BMI <18.5), 645 (76.7%) had normal weight (BMI 18.5–24.99), 141 (16.8%) were overweight (BMI 25–29.99) and 38 (4.5%) were obese (BMI ≥30) according to the classification of the World Health Organization [46]. No one had class III obesity with BMI ≥40. A very slight increase in supplemental vitD intake (0.04 μg) was observed between second and third trimester. A total of 18% followed the recommendations of a daily intake of 10 μg vitamin D supplement in the second and third trimester (Tables 1 and 2). In third trimester, 18 women had gestational hypertension and 43 had GDM. The mean BW was 3,519 ± 540 grams (S2 Table). At the second sample collection, 58 women from Trondheim and 36 from Stavanger were lost to follow-up (Fig 1). The characteristics did not differ from the original population, although a lower proportion exercised regularly pre-pregnancy (59% vs. 71%). Serum analyses from two women in second trimester and two in third trimester could not be completed in the sub-analysis (n = 250) of 1,25(OH)_2_D.

**Table 1:** Baseline demographic and clinical characteristics of the study population^†^.

Maternal characteristics	Total (*n* = 855)	Trondheim 63°N (*n* = 660)	Stavanger 58°N (*n* = 195)
Age (years)	30.5 ± 4.3	30.4 ± 4.3	30.6 ± 4.5
Gestational length at inclusion (weeks)*	20.0 ± 1.7	20.0 ± 1.7	20.7 ± 1.5
Marital status *n* (%)**			
Married/cohabitant	834 (97.7)	645 (97.9)	189 (96.9)
Single	20 (2.3)	14 (2.1)	6 (3.1)
Education level *n* (%)			
Elementary school	5 (0.6)	3 (0.5)	2 (1.0)
High School	90 (10.5)	64 (9.7)	26 (13.3)
University	760 (88.9)	593 (89.8)	167 (85.6)
Paid work or self-employed *n* (%)**	793 (92.9)	614 (93.2)	184 (94.4)
Parity *n* (%)			
0	486 (56.8)	374 (56.7)	112 (57.4)
1	254 (29.7)	199 (30.2)	55 (28.2)
2	90 (10.5)	68 (10.3)	22 (11.3)
3+	25 (2.9)	19 (2.9)	6 (3.1)
Smoking *n* (%)**	9 (1.1)	5 (0.8)	4 (2.1)
Inclusion body mass index (kg/m^2^)**	24.8 ± 3.2	24.9 ± 3.3	24.7 ± 3.0
Blood pressure (mm Hg)			
Systolic	108.9 ± 8.6	108.9 ± 8.6	108.9 ± 8.5
Diastolic	68.7 ± 7.8	69.4 ± 7.7	66.5 ± 7.7
Gestational hypertension *n* (%)^††^	9 (1.1)	8 (1.2)	1 (0.5)
Gestational diabetes *n* (%)*** ^†††^	5 (0.6)	5 (0.8)	0
Daily total vitD intake (μg)	10.4 ± 7.0	10.6 ± 7.1	9.8 ± 6.7
Daily total vitD intake <10 μg *n* (%)	507 (59.3)	383 (58.0)	124 (63.6)
Daily vitD from supplements (μg)****	5.5 ± 6.5	5.7 ± 6.6	5.0 ± 6.4
Daily intake of ≥10 μg vitD from supplements *n* (%)****	157 (18.4)	124 (18.8)	33 (17.0)
Daily intake of fish (g)****	54.8 ± 38.3	54.4 ± 38.5	56.0 ± 37.4
Intake of fish <300 g/week *n* (%)****	383 (45.0)	299 (45.4)	84 (43.3)
Daily intake of calcium (mg)****	974.8 ± 374.1	976.9 ± 373.3	967.7 ± 377.8
Daily calcium intake <900 mg *n* (%)****	401 (47.1)	304 (46.2)	97 (50.0)
Exercised regularly pre-pregnancy *n* (%)	610 (71.3)	476 (72.1)	134 (68.7)

Continuous variables are given as means ± standard deviations (SD), and categorical variables are given as (*n*) with percentages (%). The Norwegian authorities’ recommendations for pregnant women are a daily vitD supplement intake of 10 μg, a weekly intake of 300–450 g fish and additionally 900 mg calcium per day.

^†^The inclusion appointment was between 18–22 weeks of pregnancy.

^††^Gestational hypertension is defined as systolic blood pressure >140 mm Hg, diastolic blood pressure >90 mm Hg, or both in women with no pregestational hypertension.

^†††^The criteria for gestational diabetes were fasting glucose level in whole blood ≥6.1 mmol/L, or plasma glucose ≥7.0 mmol/L, or 2-hour glucose level ≥7.8 mmol/L after oral glucose tolerance test in women with no pregestational diabetes.

*Ten women from Trondheim and two from Stavanger are missing.

**One woman from Trondheim is missing.

***14 women from Trondheim and five women from Stavanger are missing.

****One woman from Stavanger and two women from Trondheim and are missing.

Abbreviation: vitD, vitamin D.

**Table 2:** Vitamin D, calcium and fish intake in third trimester*.

Variables	Total population (*n* = 761)	Trondheim 63°N (*n* = 602)	Stavanger 58°N (*n* = 159)
Daily total vitD intake (μg)	10.3 ± 7.3	10.3 ± 7.4	10.4 ± 6.9
Daily total vitD intake <10 μg *n* (%)	463 (60.8)	366 (60.8)	97 (61.0)
Daily vitD from supplements (μg)**	5.6 ± 6.8	5.6 ± 6.8	5.9 ± 6.7
Daily intake of ≥10 μg vitD from supplements *n* (%)**	139 (18.3)	108 (18.0)	31 (19.5)
Daily intake of fish (g)**	49.1 ± 32.3	49.5 ± 32.6	47.5 ± 30.9
Intake of fish <300 g/week *n* (%)**	390 (51.5)	306 (51.1)	84 (52.8)
Daily intake of calcium (mg)**	960.6 ± 344.5	962.7 ± 352.1	952.9 ± 315.1
Daily intake of calcium <900 mg *n* (%) **	348 (45.9)	276 (46.1)	72 (45.3)

Continuous variables are given as means ± standard deviations (SD) and categorical variables are given as numbers (*n*) with percentages (%). The Norwegian authorities’ recommendations for pregnant women are a daily vitD supplement intake of 10 μg, a weekly intake of 300–450 g fish and additionally 900 mg calcium per day.

*The appointment was between 32–36 weeks of pregnancy.

^**^Three from Trondheim are missing.

Abbreviation: vitD, vitamin D.

### Crude serum values in pregnancy

In Table 3 crude serum values of 25(OH)D, calculated free 25(OH)D, albumin-bound 25(OH)D, bioavailable 25(OH)D, PTH, calcium, corrected calcium, magnesium, phosphate, albumin and DBP in second and third trimester are presented. The mean crude 25(OH)D levels in second and third trimester were 66.1 ± 24.8 and 64.3 ± 27.1 nmol/L, respectively. In second trimester, 232 (27%) had vitD insufficiency and 40 (5%) deficiency. In third trimester, the corresponding numbers were 246 (34%) and 52 (7%). Mean PTH concentrations were 2.8 ± 1.09 and 3.6 ± 1.51 pmol/L, respectively, in second and third trimester. In third trimester, 27 (3.7%) had PTH levels above the upper reference limit (6.9 pmol/L). Of these, 56% had vitD insufficiency or VDD. None of those with elevated PTH had 25(OH)D levels >74 nmol/L. PTH elevation was more frequent (82%) in the autumn, winter and spring. Corrected calcium, magnesium, phosphate and creatinine levels were within reference range. In a sub-analysis, 1,25(OH)_2_D levels ranged from 97–408 and 105–408 pmol/L, in second and third trimester. Crude mean 1,25(OH)_2_D levels were 199.1 (CI 192.9 to 205.2) and 229.1 (CI 220.9 to 237.3) pmol/L in second and third trimester, respectively. Calculated free 1,25(OH)_2_D was 833.0 (CI 806.2 to 859.8) fmol/L in second trimester and 876.7 (CI 845.9 to 907.5) fmol/L third trimester.

**Table 3:** Crude serum values in second* and third** trimester.

Serum measures	Serum levels Total	Serum levels Trondheim 63°N	Serum levels Stavanger 58°N
**Second trimester**	(*n* = 855)^a^	(*n* = 660)^a^	(*n* = 195)
25OH)D (nmol/L)	66.1 ± 24.8	64.8 ± 24.2	70.4 ± 26.3
Calculated free 25(OH)D (pmol/L)^b^	15.3 ± 5.9	15.1 ± 5.8	15.9 ± 6.4
Albumin-bound 25(OH)D (nmol/L)^b^	5.06 ± 1.98	5.01 ± 1.96	5.23 ± 2.07
Bioavailable 25(OH)D (nmol/L)^b^	5.08 ± 1.99	5.03 ± 1.96	5.25 ± 2.08
PTH (pmol/L)	2.77 ± 1.09	2.82 ± 1.10	2.61 ± 1.04
Calcium (mmol/L)	2.27 ± 0.07	2.27 ± 0.07	2.27 ± 0.07
Corrected calcium (mmol/L)	2.34 ± 0.06	2.34 ± 0.06	2.34 ± 0.06
Magnesium (mmol/L)^b^	0.75 ± 0.04	0.75 ± 0.04	0.73 ± 0.05
Phosphate (mmol/L)	1.19 ± 0.12	1.20 ± 0.13	1.18 ± 0.12
Albumin (g/L)	36.7 ± 2.0	36.7 ± 2.1	36.5 ± 1.8
DBP (μmol/L)^b^	5.8 ± 0.8	5.7 ± 0.8	6.0 ± 0.8
**Third trimester**	(n = 761)	(n = 603)	(n = 158)
25OH)D (nmol/L)^c^	64.3 ± 27.1	63.6 ± 26.4	66.9 ± 29.5
Calculated free 25(OH)D (pmol/L)^d^	13.6 ± 5.8	13.6 ± 5.8	13.7 ± 6.0
Albumin-bound 25(OH)D (nmol/L)^d^	4.14 ± 1.77	4.14 ± 1.76	4.13 ± 1.81
Bioavailable 25(OH)D (nmol/L)^d^	4.15 ± 1.77	4.15 ± 1.77	4.15 ± 1.82
PTH (pmol/L)^c^	3.61 ± 1.51	3.68 ± 1.53	3.32 ± 1.40
Calcium (mmol/L)^c^	2.25 ± 0.07	2.25 ± 0.07	2.25 ± 0.07
Corrected calcium (mmol/L)^c^	2.37 ± 0.07	2.37 ± 0.07	2.38 ± 0.07
Magnesium (mmol/L)^c^	0.73 ± 0.05	0.74 ± 0.04	0.71 ± 0.04
Phosphate (mmol/L)^c^	1.17 ± 0.14	1.17 ± 0.14	1.18 ± 0.14
Albumin (g/L)^c^	33.8 ± 1.9	33.8 ± 1.9	33.6 ± 1.9
DBP (μmol/L)^d^	6.4 ± 0.9	6.3 ± 0.9	6.6 ± 0.8

Serum levels are presented as means ± standard deviations (SD).

*The blood samples were collected between 18–22 weeks of pregnancy.

**The blood samples were collected between 32–36 weeks of pregnancy.

^a^One woman from Trondheim is missing.

^b^One women from Stavanger is missing.

^c^23 women from Trondheim and 14 women from Stavanger are missing.

^d^24 women from Trondheim and 14 women from Stavanger are missing.

Abbreviations: PTH, parathyroid hormone; DBP, Vitamin D-binding protein.

### Changes in indices of vitamin D and other serum parameters between second and third trimester

A slight decrease occurred in total 25(OH)D between second and third trimester (Table 4). Increasing or indifferent levels were observed in 314 (43%), whereas 410 (57%) experienced a decline. 1,25(OH)_2_D, PTH and DBP levels were increasing, whereas calcium, magnesium, phosphate and albumin decreased (Table 4). A decline was observed in free 25(OH)D, the percentage of free 25(OH)D (0.023% versus 0.021%, p <0.0001) and bioavailable 25(OH)D. Corrected calcium was increasing (Table 4).

**Table 4:** Adjusted serum values in second and third trimester, and estimates of change between trimesters.

Serum measures	Serum values in second trimester*	Serum values in third trimester**	Estimates of change between second and third trimester (95% CI)	p-value***
25(OH)D (nmol/L)	66.4	64.6	-1.8 (-2.8 to -0.7)	0.001
Calculated free 25(OH)D (pmol/L)	15.4	13.7	-1.7 (-2.0 to -1.4)	<0.0001
Albumin-bound 25(OH)D (nmol/L)	5.09	4.15	-0.94 (-1.03 to -0.84)	<0.0001
Bioavailable 25(OH)D (nmol/L)	5.10	4.17	-0.93 (-1.03 to -0.85)	<0.0001
PTH (pmol/L)	2.78	3.59	0.81 (0.72 to 0.90)	<0.0001
Calcium (mmol/L)	2.269	2.247	-0.023 (-0.027 to -0.018)	<0.0001
Corrected Calcium (mmol/L)	2.337	2.372	0.035 (0.031 to 0.039)	<0.0001
Magnesium (mmol/L)	0.747	0.730	-0.017 (-0.020 to -0.015)	<0.0001
Phosphate (mmol/L)	1.195	1.172	-0.022 (-0.032 to -0.013)	<0.0001
Albumin (g/L)	36.7	33.7	-2.9 (-3.1 to -2.8)	<0.0001
DBP (μmol/L)	5.77	6.39	0.62 (0.57 to 0.66)	<0.0001

*The blood samples in second trimester were collected between 18–22 weeks of pregnancy.

**The blood samples in third trimester were collected between 32–36 weeks of pregnancy.

*** A mixed model with fixed effects (streg with fe option) was used in Stata. The model included a random intercept. We adjusted for season. The model-based levels in second and third trimester were estimated by using the postestimation command lincom (linear combinations of estimators).

Abbreviations: CI, confidence interval; PTH, parathyroid hormone; DBP, vitamin D- binding protein.

Among the 250 women in the sub-analysis, the seasonally adjusted 1,25(OH)_2_D concentration increased from 198.9 (CI 195.6 to 202.2) pmol/L to 230.3 (CI 226.9 to 233.8) pmol/L) (change = 31.4 (CI 24.7 to 38.2) pmol/L, p <0.0001). A decline in 1,25(OH)_2_D concentration was observed in 26% (crude data). Among those with 25(OH)D <30 nmol/L and 25(OH)D >75 nmol/L in the third trimester, 45% and 17%, respectively experienced a decline in 1,25(OH)_2_D between the trimesters. Five percent of those women with falling 1,25(OH)_2_D had PTH elevation above the reference range, and all subjects in this group showed 25(OH)D levels <34 nmol/L (third trimester). In both second and third trimester, women with vitamin D insufficiency and deficiency displayed lower 1,25(OH)_2_D levels than those with adequate vitD status. In a linear regression model, each 1 nmol/L increment in the 25(OH)D concentration increased the levels of 1,25(OH)_2_D by 0.74 pmol/L (CI 0.53 to 0.96 pmol/L, p <0.0001), and by 0.92 pmol/L (CI 0.69 to 1.14 pmol/L, p <0.0001) in second and third trimester, respectively. Season adjusted calculated free 1,25(OH)_2_D increased from 832.1 (CI 818.9 to 845.1) fmol/L in second trimester to 881.7 (CI 868.3 to 895.1) fmol/L in third trimester (change = 49.7 (CI 23.2 to 76.2) fmol/L, p <0.0001)

### Latitudinal and seasonal differences

**Fig 2:**
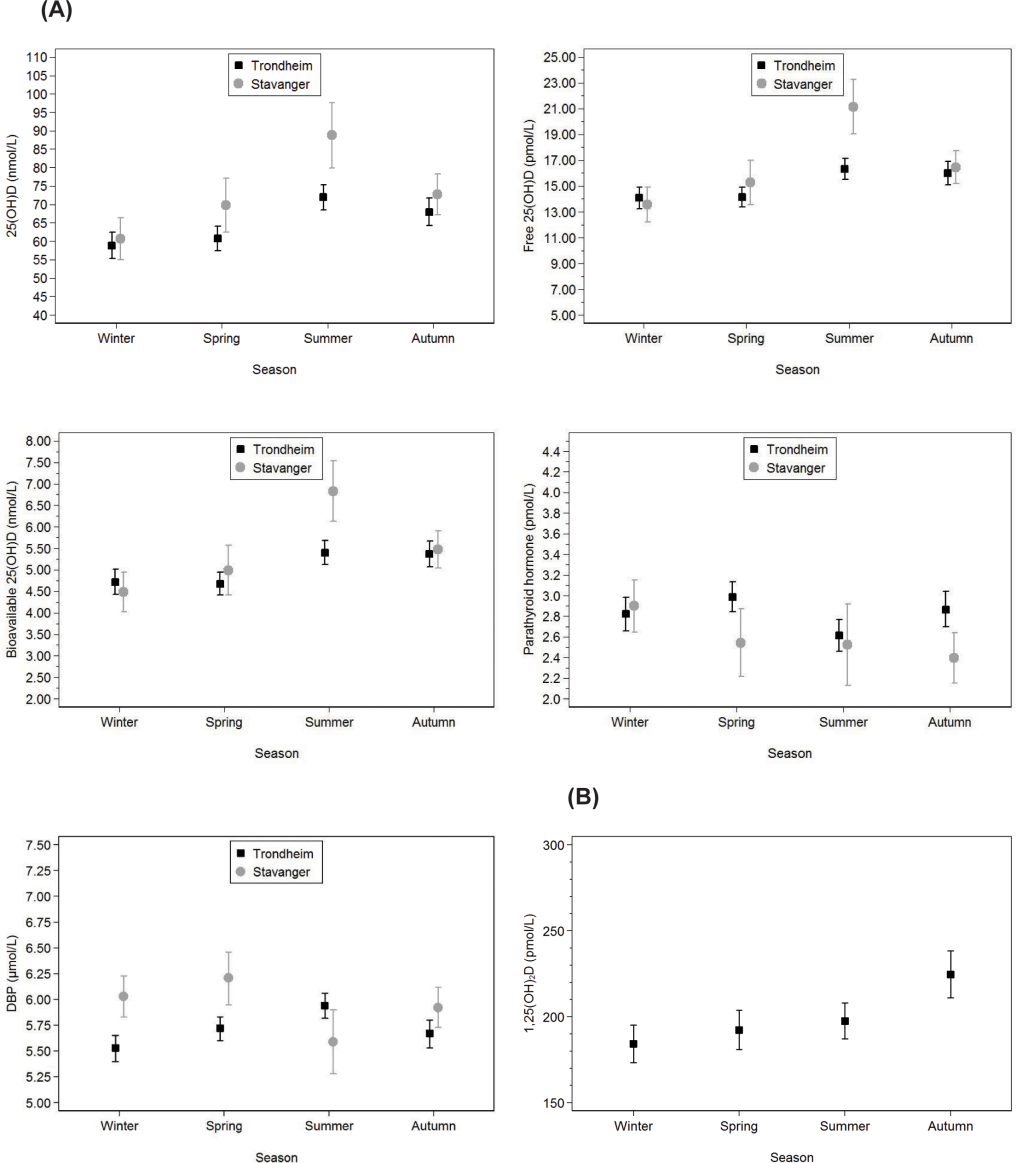
Seasonal variation of serum measures in second trimester. **(A)** Seasonal variation of serum total, free and bioavailable 25(OH)D, PTH and DBP in second trimester. **(B)** Seasonal variation of serum 1,25(OH)_2_D in second trimester, in a sub-analysis including 250 women living in Trondheim, Norway. Solid squares represent women living in Trondheim, Norway (latitude 63°N) and grey dots represent women living in Stavanger, Norway (latitude 58°N). Vertical lines represent 95% confidence intervals. A multivariable linear regression analysis was used, and separate analyses were performed for second and third trimester. In analyses involving 1,25(OH)_2_D, we used the pweight function in Stata to account for the sampling scheme (the inverse of the probability of an observation being selected into the sample). Abbreviations: PTH, parathyroid hormone; DBP, vitamin D-binding protein.

**Fig 3:**
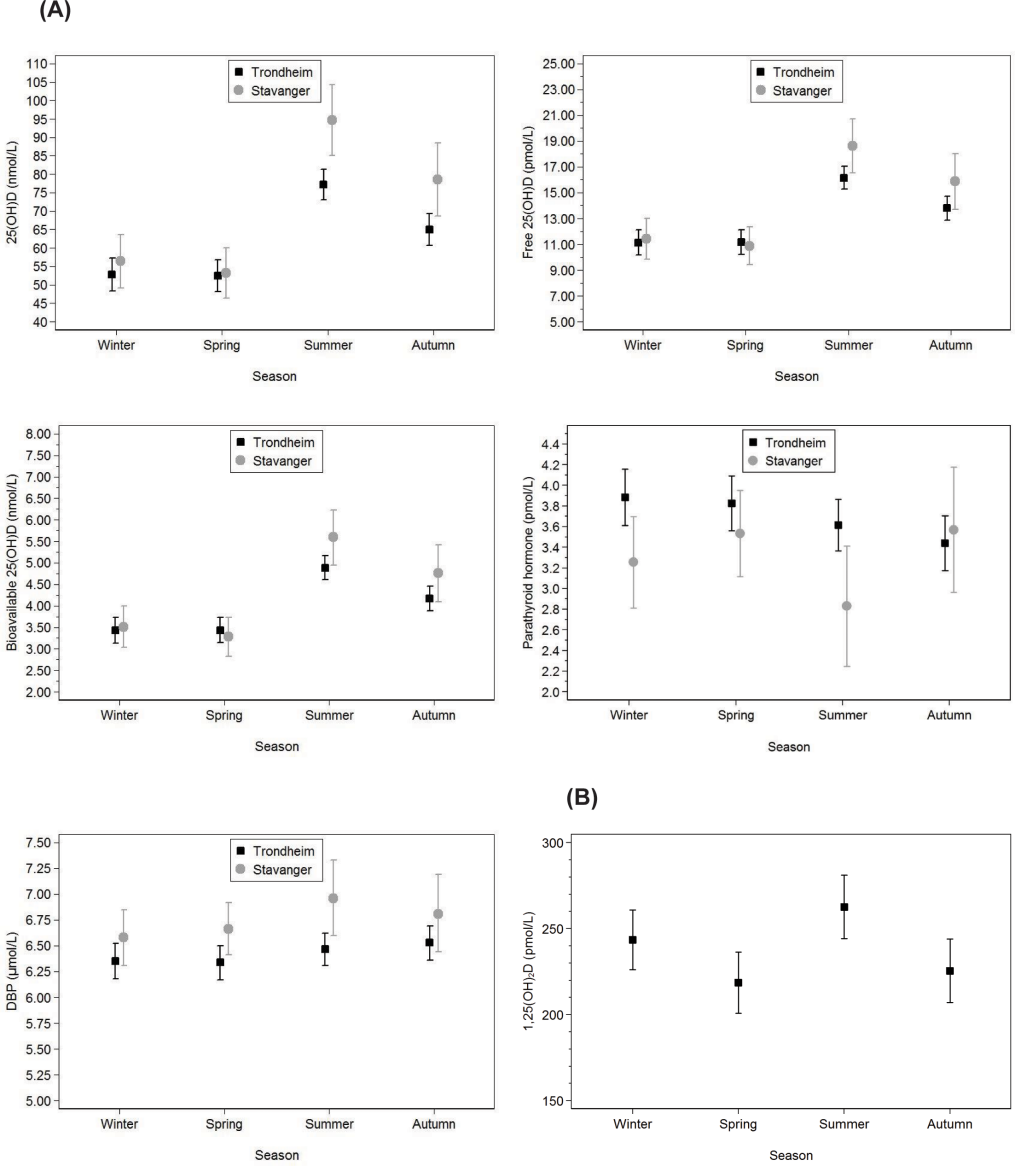
Seasonal variation of serum measures in third trimester. **(A)** Seasonal variation of serum total, free and bioavailable 25(OH)D, PTH and DBP in third trimester. **(B)** Seasonal variation of serum 1,25(OH)_2_D in third trimester, in a sub-analysis including 250 women living in Trondheim, Norway. Solid squares represent women living in Trondheim, Norway (latitude 63°N) and grey dots represent women living in Stavanger, Norway (latitude 58°N). Vertical lines represent 95% confidence intervals. A multivariable linear regression analysis was used, and separate analyses were performed for second and third trimester. In analyses involving 1,25(OH)_2_D, we used the pweight function in Stata to account for the sampling scheme (the inverse of the probability of an observation being selected into the sample). Abbreviations: PTH, parathyroid hormone, DBP, vitamin D-binding protein.

Differences in serum measures between Trondheim (latitude 63°N) and Stavanger (latitude 58°N) in second and third trimester are presented in Table 5. In both trimesters, lower levels of free and total 25(OH)D and higher PTH levels were seen at the northerly latitude. Seasonal variations in all 25(OH)D measures and PTH occurred in both trimesters, while DBP and 1,25(OH)_2_D did not show the same seasonal pattern (Figs 2 and 3). After adjustment for education and vitD supplementation, similar results were seen. In the second and third trimester, respectively, 56 (36%) and 61 (47%) from Trondheim and 25 (40%) and 23 (51%) from Stavanger exhibited vitD insufficiency during wintertime. Of the women who were in the third trimester during the dark season, 15 (12%) from Trondheim and 3 (7%) from Stavanger had VDD.

**Table 5:** Latitudinal differences between Trondheim, Norway (latitude 63°N) and Stavanger, Norway (latitude 58°N) in serum measures in second* and third trimester**.

Serum measures	Serum levels Trondheim	Serum levels Stavanger	Differences between Trondheim and Stavanger (95% CI)	p-value***
**Second trimester**	(n = 660)^a^	(n = 195)		
25(OH)D (nmol/L)	64.9	73.0	8.1 (4.2 to 12.0)	<0.0001
Calculated free 25(OH)D (pmol/L)^b^	15.1	16.6	1.5 (0.6 to 2.4)	0.002
Albumin-bound 25(OH)D (nmol/L)^b^	5.03	5.43	0.40 (0.09 to 0.71)	0.01
Bioavailable 25(OH)D (nmol/L)^b^	5.04	5.45	0.40 (0.09 to 0.72)	0.01
PTH (pmol/L)	2.82	2.59	-0.23 (-0.41 to -0.06)	0.009
Calcium (mmol/L)	2.270	2.263	-0.007(-0.019 to 0.004)	0.2
Corrected Calcium (mmol/L)	2.336	2.335	-0.001 (-0.011 to 0.009)	0.8
Magnesium (mmol/L)^b^	0.751	0.736	-0.015 (-0.022 to -0.007)	<0.0001
Phosphate (mmol/L)	1.199	1.185	-0.014 (-0.035 to 0.007)	0.2
Albumin (g/L)	36.76	36.39	-0.37 (-0.71 to -0.03)	0.03
DBP (μmol/L)^b^	5.71	5.94	0.23 (0.09 to 0.36)	0.001
**Third trimester**	(n = 603)	(n = 158)		
25(OH)D (nmol/L)^c^	61.9	70.7	8.9 (4.3 to 13.4)	<0.0001
Calculated free 25(OH)D (pmol/L)^d^	13.1	14.2	1.1 (0.2 to 2.1)	0.03
Albumin-bound 25(OH)D (nmol/L)^d^	3.969	4.271	0.302 (-0.002 to 0.606)	0.052
Bioavailable 25(OH)D (nmol/L)^d^	3.98	4.29	0.30 (-0.02 to 0.61)	0.051
PTH (pmol/L)^c^	3.69	3.30	-0.39 (-0.67 to -0.11)	0.006
Calcium (mmol/L)^c^	2.245	2.251	0.006 (-0.007 to 0.019)	0.4
Corrected Calcium (mmol/L)^c^	2.368	2.381	0.013 (0.000 to 0.025)	0.046
Magnesium (mmol/L)^c^	0.738	0.718	-0.021 (-0.029 to -0.013)	<0.0001
Phosphate (mmol/L)^c^	1.169	1.178	0.009 (-0.167 to 0.346)	0.5
Albumin (g/L)^c^	33.83	33.48	-0.35 (-0.70 to 0.00)	0.053
DBP (μmol/L)^d^	6.42	6.76	0.34 (0.16 to 0.51)	<0.0001

*The blood samples were collected between 18–22 weeks of pregnancy.

**The blood samples were collected between 32–36 weeks of pregnancy.

***A multivariable linear regression was used, and separate analyses were performed in second and third trimester.

^a^One woman from Trondheim is missing.

^b^One woman from Stavanger is missing.

^c^23 women from Trondheim and 14 women from Stavanger have missing values.

^d^24 women from Trondheim and 14 women from Stavanger have missing values.

Abbreviations: CI, confidence interval; PTH, parathyroid hormone; DBP, vitamin D-binding protein.

### Association of vitamin D measures and PTH with pregnancy outcomes (gestational diabetes mellitus and birthweight)

In the simple linear regression analysis, lower total and free 25(OH)D levels in second trimester were associated with higher BW (change in BW for each 1-unit increase in the serum measure 25(OH)D and free 25(OH)D was -1.8, (CI -3.3 to -0.4) g and -7.7 (CI -13.8 to -1.6) g, respectively). In a multivariable regression model the association was almost fully attenuated (change in BW for each 1-unit increase in the serum measure 25(OH)D and free 25(OH)D was -0.5 (CI -2.1 to 1.1) g and -1.2 (CI -7.8 to 5.4) g, respectively). No association of 1,25(OH)_2_D and PTH with BW was found in the simple linear regression analysis (change in BW for each 1-unit increase in the serum measure 1,25(OH)_2_D and PTH was 0.5 (CI -0.9 to 1.9) g and 29.2 (CI -4.2 to 62.6) g, respectively) or in a multivariable regression model (change in BW for each 1-unit increase in the serum measure 1,25(OH)_2_D and PTH was -0.3 (CI -1.8 to 1.2) g and -5.2 (CI -39.9 to 29.6) g, respectively)

No associations of 25(OH)D measures, 1,25(OH)_2_D and PTH with GDM were observed in the logistic regression modelling (25(OH)D: crude odds ratio (OR) 1.00 (CI 0.99 to 1.01), adjusted OR 1.00 (CI 0.99 to 1.02); Free 25(OH)D: crude OR 1.00 (CI 0.95 to 1.05), adjusted OR 1.00 (CI 0.94 to 1.06); 1,25(OH)_2_D: crude OR 1.00 (CI 0.99 to 1.01), adjusted OR 1.00 (CI 0.99 to 1.01); PTH: crude OR 1.19 (0.92 to 1.52), adjusted OR 1.18 (CI 0.90 to 1.54)) (S3 and S4 Tables). The same analyses were also performed in third trimester, but no substantial differences were found. After adjustment for education and intake of vitD, calcium and fish, similar results were observed.

## Discussion

To our knowledge, this is the largest longitudinal study investigating several indices of vitD metabolism at two time points during pregnancy. In accordance with previous studies, hypovitaminosis D was frequent, and 246 (34%) of the well-educated, Caucasian women had vitD insufficiency and 52 (7%) VDD in the third trimester. In spite of Northern latitudes, the prevalence was lower than reported in most previous European studies of Caucasian pregnant women [9, 16, 23, 29, 30, 47–49]. In a sub-analysis (n = 250), a decline in 1.25(OH)_2_D was observed in half of those with VDD. This was reflected in an increased occurrence of secondary hyperparathyroidism (SHPT).

Few studies have addressed free and bioavailable 25(OH)D during pregnancy [21, 23, 24]. We measured DBP concentrations at two time points which allowed us to calculate free and bioavailable 25(OH)D [41, 42]. Schwartz et al. observed similar measured free 25(OH)D concentrations in pregnant women and a comparator group [24]. A reference range for directly measured free 25(OH)D (5.3–7.7 pg/mL ≈ 13.1–19.3 pmol/L) was provided in a recent study [22]. The levels were in the same range in our study subjects (13.6–15.3 pmol/L), although calculated free 25(OH)D has been claimed to overestimate the level [24, 50]. In accordance with previous studies, a rise occurred in DBP levels, which contribute to the decline in free and bioavailable 25(OH)D [21, 23]. A Korean study showed lower levels of calculated bioavailable 25(OH)D in pregnant than in non-pregnant women (median 1.7 ng/mL ≈ 4.3 nmol/L in second and third trimester), whereas total levels were indifferent [21]. Median level of calculated bioavailable 25(OH)D was similar (4.4 nmol/L) in the current study. Testing of both total, free and bioavailable 25(OH)D would provide a better assessment of vitD status in conditions with changes in DBP levels like pregnancy.

Consistent with previous studies, a rise occurred in 1,25(OH)_2_D levels between second and third trimester [23, 26–28]. We also calculated free 1,25(OH)_2_D levels, since the free hormone is responsible for the biological actions, and observed an increment in correspondence with total 1,25(OH)_2_D [14, 22, 44, 51, 52].

A decrease in 1,25(OH)_2_D levels was, however, found in 45% of those with VDD in the final trimester. These women also displayed lower 1,25(OH)_2_D levels in both trimesters compared to those with circulating 25(OH)D >75 nmol/L. Recently, Hollis et al. suggested that maximal 1,25(OH)_2_D concentrations during pregnancy require 25(OH)D levels of 100 nmol/L [28]. In the current study, only 10% of the total population reached this threshold. Most studies of 1,25(OH)_2_D levels in pregnancy have a small sample size, and show a large variation in third trimester (mean range 86.4–283.0 pmol/L) [18]. In comparison, we observed 1,25(OH)_2_D concentration within the upper range (229.1 pmol/L), ranging from 105 to 408 pmol/L. The intestinal calcium absorption doubles during pregnancy, mainly attributed to the increase in 1,25(OH)_2_D [4, 26, 27, 51, 53]. The rise in 1,25(OH)_2_D is not driven by PTH, as a decline to the lower end of the reference level occurs [4, 26, 27]. Thus, other regulators of 1-alpha-hydroxylase as parathyroid hormone-related protein (PTHrP), placental lactogen and estradiol must account for most of the circulating 1,25(OH)_2_D during pregnancy [8, 26, 27]. Prolactin and placental lactogen have been proposed to compensate for the lack of vitD [26]. PTHrP, which peaks late in pregnancy, could be involved in keeping serum calcium at an adequate level by mobilizing calcium from bone [26]. In the current study, 4% exhibited elevated PTH levels, preferentially in months with little UVB radiation. Of these, 56% had vitD insufficiency consistent with SHPT. A similar prevalence (2%) of SHPT among Caucasian women was found in a UK study [54]. In the current study, PTH elevation was not seen in women with 25(OH)D levels above 74 nmol/L. This complies with Kramer et al. who reported PTH suppression at 25(OH)D levels >82 nmol/L during pregnancy [55]. The corresponding level in the non-pregnant state was 81 nmol/L [55]. This indicates similar thresholds for vitD supplementation in pregnancy as in the non-pregnant state. This is in line with the classification of the Endocrine Society (vitD insufficiency <75 nmol/L) [3, 17].

The significance of the low levels of 1,25(OH)_2_D, as noticed in a proportion of our study subjects, is little explored. A concomitant rise in PTH and PTHrP at the end of pregnancy may pose a substantial burden on the maternal skeleton and could explain some of the cases with pregnancy-associated osteoporosis. Although 1,25(OH)_2_D is replaced by compensatory hormones to maintain calcium homeostasis, we postulate that this could impact the fetal and maternal skeleton adversely.

So far, the relationship between maternal 1,25(OH)_2_D levels and skeletal outcomes has not been addressed, while studies on 25(OH)D status and bone health in the offspring are diverging [8, 27]. Severe VDD in pregnancy is associated with hypocalcemia, rickets and craniotabes in the infant [4, 8, 10, 15, 17, 27]. Observational studies show a positive association between maternal calcium intake and fetal bone development, as well as postnatal bone mineral content (BMC) and bone mineral density (BMD), while RCTs show conflicting results [53]. In a RCT from the UK, a daily supplementation of 25 μg vitD during pregnancy did not improve BMC of the infant [56]. However, in a subgroup born in the winter season, a significant effect on BMC was seen [56]. Longitudinal studies investigating association between maternal vitD status and BMC in offspring at 9-years of age show diverging results [8, 57]. In an Australian study (n = 341 mother-offspring pairs), maternal vitD inadequacy in second trimester was associated with reduced peak bone mass in the 20-year-old offspring, implying increased risk for osteoporosis in the future [58].

Numerous studies, including a meta-analysis have shown that VDD is associated with adverse pregnancy outcomes [1, 4, 6, 7]. The meta-analysis of observational studies, which included more than 22,000 women, concluded that maternal VDD is associated with an increased risk for GDM and lower birthweight infants [6]. A Canadian study demonstrated an association between PTH and GDM, but not 25(OH)D and 1,25(OH)_2_D, and pregnancy outcomes [59]. We found no association of vitD measures and PTH with GDM. In contrast to other studies, lower 25(OH)D was related to higher BW, but not after adjusting for potential confounding factors [6, 27]. The same relationship was observed between free 25(OH)D and BW. In previous studies reporting a positive association between maternal vitD and birthweight, a higher proportion of women had VDD compared to our study [60, 61]. Hollis et al. proposed that 25(OH)D levels should be at least 100 nmol/L to give beneficial health effects and lower risk for adverse pregnancy outcomes [20, 52, 62]. This was supported by a recent study showing that pregnant women with 25(OH)D levels ≥100 nmol/L had a 62% lower risk of preterm birth compared to those with concentrations <50 nmol/L [62]. The fact that only 10% of our study participants reached levels ≥100 nmol/L may have reduced our ability to detect an association. Neither 1,25(OH)_2_D nor PTH were associated with BW. Morley et al. reported a positive relationship between PTH and BW, whereas 25(OH)D was associated with reduced intrauterine long bone growth [63]. RCTs addressing the effects of vitD supplementation on pregnancy outcomes have shown diverging results [1, 7, 8, 27, 54]. This may be attributed to differences between studies, including the prevalence of VDD, calcium status, the vitD supplement dose, and start of the intervention [4, 7, 8, 27].

Several factors influence 25(OH)D levels including ethnicity, food and sun habits, latitude, altitude, season, and genetic polymorphisms [15–17, 29, 30]. Most previous studies show minor changes of 25(OH)D during pregnancy [16, 23, 26]. This is in line with our findings showing a very modest decline in 25(OH)D between second and third trimester. Maternal 25(OH)D levels seem to remain relatively stable during pregnancy, despite the increased synthesis of 1,25(OH)_2_D and the transplacental transfer of 25(OH)D to the fetus [26]. Severe VDD during pregnancy was observed in a small proportion of the study subjects. This is of concern, as normal vitD levels in the neonates are reliant on adequate maternal vitD status [64].

Few studies have addressed vitD status in pregnant women at northern latitudes and the impact of small latitudinal differences [47]. Despite minor differences in latitude and similar intake of vitD, levels of total and free 25(OH)D were lower at the northerly study site in both trimesters. VitD insufficiency was less frequent in third trimester (34%) than in a Swedish study (65%), performed at the same latitude as our southern study site [30]. This was reflected in higher mean PTH level among the Swedish women [30]. The difference in prevalence may be attributed to lower vitD intake in the Swedish study.

In the dark season, UVB-mediated synthesis of vitD is absent at northern latitudes, and vitD has to be obtained through diet and supplements [9, 15, 47]. Western-style diet has low content of vitD, and in Norway, few foods are fortified with vitD [17, 65]. In Norway, the authorities recommend a daily vitD supplement of 10 μg, a weekly intake of 300–450 g fish, and additionally 900 mg calcium daily [25, 66]. In contrast, there are no specific Swedish supplement recommendations [30, 67]. It is of concern that only 18% of the well-educated women followed the vitD supplement advices, and only half adhered to the recommendations concerning fish and calcium intake.

The major strengths of the present study are the large number of participants recruited all year round, a high follow-up rate, repeated sampling during pregnancy and standardized procedures for sampling. Analyses were performed concurrently, applying the same instruments and procedures. The study population was ethnically homogenous contributing to limited bias due to skin pigmentation, clothing habits and genetic polymorphisms [7, 8, 15–17]. Furthermore, the study sites were located in different geographical regions of Norway, providing an opportunity to investigate latitudinal differences.

The participants were well-educated Caucasian women with low-risk pregnancies, which may affect the generalizability. Serum 25(OH)D was analyzed by ECLIA (Roche), although liquid chromatography-tandem mass spectrometry (LC-MS/MS) is considered to be the golden standard [17]. The FFQ used in this study may overestimate the intake of vitD [35]. The calculation of free 25(OH)D and 1,25(OH)D are dependent on several factors, including accurate measurements of DBP and albumin [44, 50]. Nielsen et al. reported a high correlation between calculated free and directly measured 25(OH)D (r = >0.80), albeit calculation of free 25(OH)D levels may give an overestimation compared to direct measurement [24, 50]. In this study, several comparisons were made, thus there is an increased probability for false positive findings, and the results need to be interpreted with care.

## Conclusions

Although Norwegian authorities recommend vitD supplementation and fish intake during pregnancy, we show that hypovitaminosis D in pregnancy is frequent in well-educated Caucasian women, particularly during wintertime. This was reflected in low adherence to the recommendations. Despite minor differences in latitude, levels of total and free 25(OH)D were lower at the northerly study sight at both second and third trimester. It is noticeable that almost half of those with 25(OH)D levels below 30 nmol/L experienced a decline in 1,25(OH)_2_D concentration between second and third trimester (sub-analysis). These women also displayed lower 1,25(OH)_2_D levels, as reflected in PTH elevation. The current findings are of concern as maternal vitD insufficiency has been shown to associate with lower offspring peak bone mass. Our data highlight the need for increased attention regarding vitD requirement during pregnancy among policy-makers, physicians and the general population. The authorities’ recommendations should be revisited, and strategies to ensure adherence should be implemented.

## Supporting information

S1 Table The reference range, limit of detection and total analytical coefficient of variation (CV) of biochemical methods used. *25(OH)D, PTH, total calcium, magnesium, phosphate, albumin and creatinine were analyzed at Department of Medical Biochemistry, St. Olavs hospital, Trondheim University Hospital.
**DBP and 1,25(OH)_2_D were analyzed at Hormone Laboratory, Oslo University Hospital. Abbreviations: CV, total analytical coefficient of variation; PTH, parathyroid hormone; ECLIA, electrochemiluminescence immunoassay; DBP, Vitamin D-binding protein; RIA, radioimmunoassay.
(DOCX)

S2 Table Pregnancy outcomes. Continuous variables are given as means ± standard deviations (SD) and categorical variables are given as numbers (*n*) with percentages (%).
†Gestational hypertension was defined as systolic blood pressure >140 mm Hg, diastolic blood pressure >90 mm Hg, or both in women with no pregestational hypertension.
††The criteria for gestational diabetes were fasting glucose level in whole blood ≥6.1 mmol/L,or plasma glucose ≥7.0 mmol/L, or 2-hour glucose level ≥7.8 mmol/L after oral glucose tolerance test in women with no pregestational diabetes.
*A total of 81 women from Trondheim and 49 from Stavanger are missing.
**A total of 100 women from Trondheim and 53 women from Stavanger are missing.
***A total of 60 women from Trondheim and 10 women from Stavanger are missing.
****Two women from Trondheim and one from Stavanger are missing.
*****One woman from Trondheim and one from Stavanger are missing.
(DOCX)

S3 Table Association of vitamin D measures and PTH with birthweight. Linear regression analysis was used to estimate the change of birthweight for each 1-unit increase in the serum measure.
^†^The blood samples were collected in second trimester (pregnancy week 18–22).
*In this analysis, we adjusted for study site, season, age, pre-pregnancy BMI, parity and pre-pregnancy physical activity.
**In a sub-analysis of 1,25(OH)_2_D, 250 women from Trondheim were included. We have applied probability weights (the inverse of the probability of an observation being selected into the sample) in the statistical analysis of 1,25(OH)_2_D to produce estimates representative of the total Trondheim population.
Abbreviations: PTH, parathyroid hormone, CI, confidence interval; BMI, body mass index.
(DOCX)

S4 Table Association of vitamin D measures and PTH with gestational diabetes mellitus. Logistic regression analysis was used to estimate odds ratio. Odds ratio for gestational diabetes mellitus for each 1-unit increase in the serum measure.
^†^The blood samples were collected in second trimester (pregnancy week 18–22).
*In this analysis, we adjusted for study site, season, age, pre-pregnancy BMI, parity and pre-pregnancy physical activity.
**In a sub-analysis of 1,25(OH)_2_D, 250 women from Trondheim were included. We have applied probability weights (the inverse of the probability of an observation being selected into the sample) in the statistical analysis of 1,25(OH)_2_D to produce estimates representative of the total Trondheim population.
Abbreviations: PTH, parathyroid hormone; OR, Odds ratio; CI, Confidence Interval; BMI, body mass index.
(DOCX)
