# Supplementary material for: Alterations in the vitamin D endocrine system during pregnancy: A longitudinal study of 855 healthy Norwegian women
Source: PLoS One. 2018 Apr 11;13(4):e0195041. doi: 10.1371/journal.pone.0195041 (PMC5895009; doi:10.1371/journal.pone.0195041)
Supplement: S1 Table — *25(OH)D, PTH, total calcium, magnesium, phosphate, albumin and creatinine were analyzed at Department of Medical Biochemistry, St. Olavs hospital, Trondheim University Hospital. **DBP and 1,25(OH)2D were analyzed at Hormone Laboratory, Oslo University Hospital. Abbreviations: CV, total analytical coefficient of variation; PTH, parathyroid hormone; ECLIA, electrochemiluminescence immunoassay; DBP, Vitamin D-binding protein; RIA, radioimmunoassay. (DOCX) [file pone.0195041.s001.docx]

**S1 Table.** **The reference range, limit of detection and total analytical coefficient of variation (CV) of biochemical methods used**

| **Serum measure** | **Method for measurement**  (manufacturer) | **Total analytical coefficient of variation** (CV) | **The limit of detection** | **Reference range** |
| --- | --- | --- | --- | --- |
| Total 25(OH)D (nmol/L)^*^ | ECLIA  (Roche Diagnostics Ltd) | 8.6% at 70 nmol/L | 8 nmol/L | 45-161 nmol/L |
| PTH (pmol/L)^*^ | ECLIA  (Roche Diagnostics Ltd) | 3.5% at 2.7 pmol/L | 0.1 pmol/L | 1.6-6.9 pmol/L |
| Total calcium (mmol/L)^*^ | Colorimetric method  (Roche Diagnostics Ltd) | 1.5% at 2.6 mmol/L | 0.20 mmol/L | 2.15-2.51 mmol/L |
| Magnesium (mmol/L)^*^ | Photometric method  (Roche Diagnostics Ltd) | 1.6% at 0.43 mmol/L | 0.03 mmol/L | 0.71-0.94 mmol/L |
| Phosphate (mmol/L)^*^ | Photometric method  (Roche Diagnostics Ltd) | 1.9% at 1.1 mmol/L | 0.1 mmol/L | 0.85-1.50 mmol/L |
| Albumin (g/L)^*^ | Photometric method  (Roche Diagnostics Ltd) | 1.7% at 45 g/L | 5 g/L | 18-39 years old: 36-48 g/L  40-46 years old: 36-45 g/L |
| Creatinine (μmol/L)^*^ | Photometric method  (Roche Diagnostics Ltd) | 1.8% at 156 µmol/L | 5 µmol/L | 45-90 µmol/L |
| DBP (μmol/L)^**^ | RIA with polyclonal antibody (Hormone Laboratory, Oslo University Hospital) | 13% at 6.8 μmol/L | 0.7 μmol/L | 2. trimester: 4.6-11.28 µmol/L 3. trimester: 3.38-12.9 µmol/L |
| 1,25(OH)_2_D (pmol/L)^**^ | Enzyme immunoassay (IDS Nordic A/S immunodiagnosticsystems) | 18.2% at 82 pmol/L | 14 pmol/L | 39-193 pmol/L |

^*^25(OH)D, PTH, total calcium, magnesium, phosphate, albumin and creatinine were analyzed at Department of Medical Biochemistry, St. Olavs hospital, Trondheim University Hospital.

^**^DBP and 1,25(OH)_2_D were analyzed at Hormone Laboratory, Oslo University Hospital.

Abbreviations: CV, total analytical coefficient of variation; PTH, parathyroid hormone; ECLIA, electrochemiluminescence immunoassay; DBP, Vitamin D-binding protein; RIA, radioimmunoassay.
