# Supplementary material for: Alterations in the vitamin D endocrine system during pregnancy: A longitudinal study of 855 healthy Norwegian women
Source: PLoS One. 2018 Apr 11;13(4):e0195041. doi: 10.1371/journal.pone.0195041 (PMC5895009; doi:10.1371/journal.pone.0195041)
Supplement: S2 Table — Continuous variables are given as means ± standard deviations (SD) and categorical variables are given as numbers (n) with percentages (%). †Gestational hypertension was defined as systolic blood pressure >140 mm Hg, diastolic blood pressure >90 mm Hg, or both in women with no pregestational hypertension. ††The criteria for gestational diabetes were fasting glucose level in whole blood ≥6.1 mmol/L,or plasma glucose ≥7.0 mmol/L, or 2-hour glucose level ≥7.8 mmol/L after oral glucose tolerance test in women with no pregestational diabetes. *A total of 81 women from Trondheim and 49 from Stavanger are missing. **A total of 100 women from Trondheim and 53 women from Stavanger are missing. ***A total of 60 women from Trondheim and 10 women from Stavanger are missing. ****Two women from Trondheim and one from Stavanger are missing. *****One woman from Trondheim and one from Stavanger are missing. (DOCX) [file pone.0195041.s002.docx]

**S2 Table. Pregnancy outcomes**

| **Pregnancy outcomes** | **Total**  (*n* = 855) | **Trondheim**  (*n* = 660) | **Stavanger**  (*n* =195) |
| --- | --- | --- | --- |
| Gestational hypertension in third trimester^*^† | 18 (2.5) | 15 (2.6) | 3 (2.1) |
| Gestational diabetes mellitus in third trimester^**^†† | 43 (6.1) | 34 (6.1) | 9 (6.3) |
| Caesarean delivery^***^ | 85 (10.9) | 63 (10.5) | 22 (12.3) |
| Gestational age at birth (days)^****^ | 280 ± 18 | 280 ± 19 | 281 ± 13 |
| Birth weight (g)^*****^ | 3,519 ± 540 | 3,519 ± 533 | 3,517 ± 564 |
| Birth weight at least 4,000 g^*****^ | 149 (17.5) | 113 (17.1) | 36 (18.6) |
| Birth weight <2,500 g^*****^ | 30 (3.5) | 23 (3.5) | 7 (3.6) |

Continuous variables are given as means ± standard deviations (SD) and categorical variables are

given as numbers (*n*) with percentages (%).

†Gestational hypertension was defined as systolic blood pressure >140 mm Hg, diastolic blood

pressure >90 mm Hg, or both in women with no pregestational hypertension.

††The criteria for gestational diabetes were fasting glucose level in whole blood >6.1 mmol/L,

or plasma glucose >7.0 mmol/L, or 2-hour glucose level >7.8 mmol/L after oral glucose tolerance test in women with no pregestational diabetes.

^*^A total of 81 women from Trondheim and 49 from Stavanger are missing.

^**^A total of 100 women from Trondheim and 53 women from Stavanger are missing.

^***^A total of 60 women from Trondheim and 10 women from Stavanger are missing.

^****^Two women from Trondheim and one from Stavanger are missing.

^*****^One woman from Trondheim and one from Stavanger are missing.
