# Supplementary material for: Alterations in the vitamin D endocrine system during pregnancy: A longitudinal study of 855 healthy Norwegian women
Source: PLoS One. 2018 Apr 11;13(4):e0195041. doi: 10.1371/journal.pone.0195041 (PMC5895009; doi:10.1371/journal.pone.0195041)
Supplement: S4 Table — Logistic regression analysis was used to estimate odds ratio. Odds ratio for gestational diabetes mellitus for each 1-unit increase in the serum measure. †The blood samples were collected in second trimester (pregnancy week 18–22). *In this analysis, we adjusted for study site, season, age, pre-pregnancy BMI, parity and pre-pregnancy physical activity. **In a sub-analysis of 1,25(OH)2D, 250 women from Trondheim were included. We have applied probability weights (the inverse of the probability of an observation being selected into the sample) in the statistical analysis of 1,25(OH)2D to produce estimates representative of the total Trondheim population. Abbreviations: PTH, parathyroid hormone; OR, Odds ratio; CI, Confidence Interval; BMI, body mass index. (DOCX) [file pone.0195041.s004.docx]

**S4 Table. Association of vitamin D measures and PTH with gestational**

**diabetes mellitus**

|  |  | **Unadjusted model** | | |  | **Adjusted model*** | | |
| --- | --- | --- | --- | --- | --- | --- | --- | --- |
| **Serum measure**^†^ |  | **OR** | **95% CI** | **p-value** |  | **OR** | **95% CI** | **p-value** |
| 25(OH)D (nmol/L) |  | 1.00 | 0.99 to 1.01 | 0.699 |  | 1.00 | 0.99 to 1.02 | 0.600 |
| Calculated free 25(OH)D  (pmol/L) |  | 1.00 | 0.95 to 1.05 | 0.889 |  | 1.00 | 0.94 to 1.06 | 0.899 |
| 1,25(OH)_2_D (pmol/L)******  PTH (pmol/L) |  | 1.00  1.19 | 0.99 to 1.01  0.92 to 1.52 | 0.497  0.185 |  | 1.00  1.18 | 0.99 to 1.01  0.90 to 1.54 | 0.390  0.226 |

Logistic regression analysis was used to estimate odds ratio. Odds ratio for gestational diabetes mellitus for each 1-unit increase in the serum measure.

^†^The blood samples were collected in second trimester (pregnancy week 18-22).

*In this analysis, we adjusted for study site, season, age, pre-pregnancy BMI, parity and pre-pregnancy physical activity.

**In a sub-analysis of 1,25(OH)_2_D, 250 women from Trondheim were included. We have applied probability weights (the inverse of the probability of an observation being selected into the sample) in the statistical analysis of 1,25(OH)_2_D to produce estimates representative of the total Trondheim population.

Abbreviations: PTH, parathyroid hormone; OR, Odds ratio; CI, Confidence Interval; BMI, body mass index
